# Hox gene regulation in the central nervous system of *Drosophila*

**DOI:** 10.3389/fncel.2014.00096

**Published:** 2014-04-23

**Authors:** Maheshwar Gummalla, Sandrine Galetti, Robert K. Maeda, François Karch

**Affiliations:** ^1^Department of Genetics and Evolution, University of GenevaGeneva, Switzerland; ^2^Institute of Biochemistry, University of Medicine – University of GöttingenGöttingen, Germany

**Keywords:** Hox genes, *abd-A*, ncRNA, miRNA, bithorax-complex

## Abstract

Hox genes specify the structures that form along the anteroposterior (AP) axis of bilateria. Within the genome, they often form clusters where, remarkably enough, their position within the clusters reflects the relative positions of the structures they specify along the AP axis. This correspondence between genomic organization and gene expression pattern has been conserved through evolution and provides a unique opportunity to study how chromosomal context affects gene regulation. In *Drosophila,* a general rule, often called “posterior dominance,” states that Hox genes specifying more posterior structures repress the expression of more anterior Hox genes. This rule explains the apparent spatial complementarity of Hox gene expression patterns in *Drosophila*. Here we review a noticeable exception to this rule where the more-posteriorly expressed *Abd-B* Hox gene fails to repress the more-anterior *abd-A* gene in cells of the central nervous system (CNS). While *Abd-B* is required to repress ectopic expression of *abd-A* in the posterior epidermis, *abd-A* repression in the posterior CNS is accomplished by a different mechanism that involves a large 92 kb long non-coding RNA (lncRNA) encoded by the intergenic region separating *abd-A* and *Abd-B* (the iab8ncRNA). Dissection of this lncRNA revealed that *abd-A* is repressed by the lncRNA using two redundant mechanisms. The first mechanism is mediated by a microRNA (mir-iab-8) encoded by intronic sequence within the large *iab8*-ncRNA. Meanwhile, the second mechanism seems to involve transcriptional interference by the long iab-8 ncRNA on the *abd-A* promoter. Recent work demonstrating CNS-specific regulation of genes by ncRNAs in *Drosophila,* seem to highlight a potential role for the *iab-8-*ncRNA in the evolution of the *Drosophila* Hox complexes.

## Hox CLUSTERS

Hox genes specify the structures that form along the anteroposterior (AP) axis of bilateria. They are strikingly conserved between invertebrates and vertebrates. This conservation extends past the gene sequences and into their relative positioning along the chromosome, as Hox genes are generally found in clusters (or complexes) in which the individual Hox genes are aligned along the chromosome in the same order as the structures they specify along the AP axis (McGinnis and Krumlauf, 1992). While this correspondence between genomic organization and body axis is suggestive of a fundamental mechanism of activation that has been conserved through evolution, thus far, no common overlying principle can completely explain the evolutionary conservation of the collinear alignment of the genes. In fact, clustering does not seem to be absolutely necessary for proper Hox gene regulation in *Drosophila,* the place where Hox genes were first discovered. Indeed, the Hox gene cluster in fruit flies has been split at different location during the evolution of the *Drosophila* lineage (Lewis et al., 2003; Negre et al., 2003; Negre and Ruiz, 2007). In *D. melanogaster*, the Hox genes have been split into two clusters separated between the *Antennapedia* (*Antp*) and *Ultrabithorax* (*Ubx*) Hox genes (forming the *Antp* complex, and the bithorax complex, BX-C). Meanwhile, in *Drosophila virilis*, the complex is split between the *Ubx* and *abd-A* genes (Von Allmen et al., 1996). However, the fact that the *Drosophila* Hox complex has been split does not mean that the remaining collinear arrangement of the *Drosophila* Hox genes plays no role in their regulation. In fact, based on genetic rearrangement experiments, we know that the collinear arrangement of the *Drosophila* Hox genes is important for their proper expression (Maeda and Karch, 2010). Thus, the breaks found in the *Drosophila* Hox complexes may be exceptional cases of rearrangements that bypassed deleterious effects.

Based on our current understanding of Hox gene regulation in vertebrates and invertebrates, it now seems likely that at least some of the reason for preserving collinearity diverged during the evolutionary history of the two lineages. In mammals, collinearity seems to be preserved primarily due to the sharing of distal enhancer elements. Within the mouse Hoxd cluster, for example, it has been shown that Hox gene expression is controlled by shared remote enhancers located, 5′ and 3′ to the Hox complex. This sharing of enhancers presumably provides evolutionary pressure to keep the Hox genes clustered. Furthermore, it seems that distance from these enhancers controls the timing and ultimate location of Hox gene expression, providing pressure to preserve collinearity. However, this is not the case in invertebrates. In *Drosophila*, Hox gene expression is controlled by gene-specific enhancers located within the complex itself. It is perhaps for this reason that invertebrate Hox complexes are generally larger than their vertebrate counterparts and why the *Drosophila* Hox complex could be split in two.

Work on non-coding RNAs (ncRNAs) has provided an additional aspect regarding the conservation of the Hox gene clusters. Two microRNA genes (miRNA) have been found at similar positions within the Hox clusters of vertebrates and arthropods (Lagos-Quintana et al., 2003). The conserved miR-10 miRNA lies between the *Drosophila* Hox genes *Deformed* and *Sex-comb-reduced*. These fly Hox genes correspond to mammalian orthologs *Hox4* and *Hox5*, respectively. Remarkably, the vertebrate miR-10b miRNA can be found between the *Hox4* and *Hox5* paralogs in the* HoxB* complex. A second miRNA gene in vertebrates (miR-196) is located between the *Hox9* and *Hox10* paralogs in the *HoxA* complex. These genes correspond to the fly genes *abd-A* and *Abd-*B. As in the case of miR10, a miRNA gene is found at a similar location in arthropods, though the primary sequence of the miRNA genes differ between the two lineages. In *Drosophila*, this miRNA gene is transcribed on both strands, giving rise to *miR-iab-4* on one strand, and *miR-iab-8* on the other strand. The *miR-iab-8* template is embedded in a very large transcription unit of >92 kb (the iab-8ncRNA). Recent work from our lab on the *iab-8*-ncRNA has led to a number of interesting results, and provide additional reasons for the preservation of Hox clustering.

## THE BITHORAX COMPLEX

**FIGURE 1:**
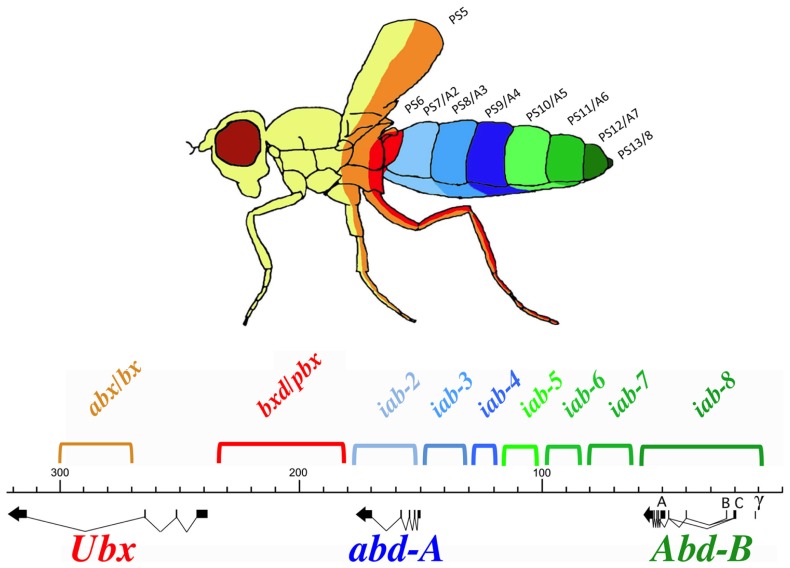
**Synopsis of the BX-C.** The genomic region of the BX-C is marked off in kilobases according to the numbering of (Martin et al., 1995). The three transcription units *Ubx*, *abd-A,* and *Abd-B* with their exons marked as thick lines and the arrows showing the transcription polarity are drawn below the DNA map. The horizontal and colored brackets above the DNA line indicate the extends of the segment-specific *cis-* regulatory regions with the following color code. Orange and red (*abx/bx* and *bxd/pb*) regulate expression of *Ubx* in PS5/T3 and PS6/A1, respectively. The blue *iab-2, iab-3,* and *iab-4* regions regulate *abd-A* expression in PS7/A2, PS8/A3, and PS9/A4. Finally, the green *iab-5, iab-6, iab-7,* and *iab-8* regulate *Abd-B* expression in PS10/A5, PS11/A6, PS12/A7, and PS13/A8, respectively. These segmental boundaries are depicted with the same colors on the fly above the BX-C map. Note that the parasegmental boundaries are visible in the thoracic segments where PS5 corresponds to the posterior part of T2 and the anterior part of T3. PS6 corresponds to posterior T3 and anterior A1.

**FIGURE 2:**
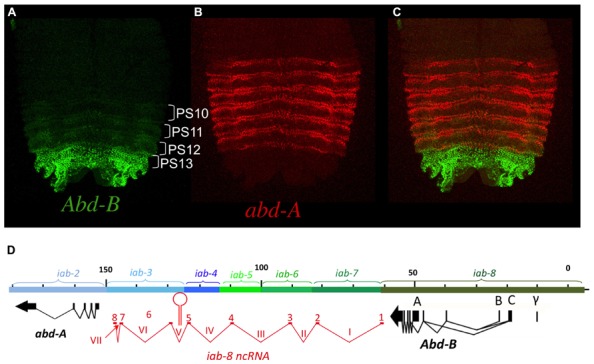
***abd-A* and *Abd-B* are expressed in broad domains.** Panels **A**, **B**, and **C** show pelts of stage 13 embryos. In these preparations, embryos were cut along the dorsal midline and flattened on a slide. Anterior is at the top. In stage 13 embryos, Hox gene expression is mostly visible in the epidermis with *abd-A* displayed in red and *Abd-B* in green. In panel **A**, *Abd-B* appears in a graded fashion from PS10 to PS13 (parasegments are marked by brackets). In these parasegments, *Abd-B* is produced from promoter A under the regulation of, respectively the *iab-5, iab-6, iab-7,* and *iab-8* regulatory regions (see also text). In PS14 an alternative form of *Abd-B* is produced from promoters B, C, and γ. The *abd-A* expression pattern in PS7 to PS12 is shown in panel **B**. Both *abd-A* and *Abd-B* are displayed in panel **C**. Note that their overall expression domains appear complementary to each other. Original observations published in (Celniker et al., 1990; Karch et al., 1990; Gummalla et al., 2012) The abdominal part of the BX-C is shown in panel **D** with the same map coordinates as in **Figure 1** (Martin et al., 1995) and with the same color code for the *abd-A, Abd-B* genes and their respective regulatory domains. The structure of the *iab-8 ncRNA* is shown in red under te DNA map. Introns are numbered with latin numbers and exons with regular numbering. Note that the polarity of transcription is the same as that for *abd-A* and *Abd-B*. Note also the presence of one exon for each of the *iab cis*-regulatory regions to the exception of 2 exons in *iab-3*. The location of miRiab-4/iab-8 in intron V is shown.

Hox genes were discovered through mutations that affect the identities of the segments that form along the AP axis of the fly. Many of these mutations were identified within the posterior Hox complex of the fly, called the BX-C (Lewis, 1978 for review, see Maeda and Karch, 2006). The BX-C encodes three Hox genes, *Ubx*, *abd-A*, and *Abd-B* (**Figure 1**), which are responsible for the identities of parasegments 8 to 13. These parasegments form the posterior thorax and all the abdominal segments of the fly (posterior T2, T3 and all eight abdominal segments A1–A8)^1^. Before the molecular genetic era, classical genetic analysis revealed the existence of mutations that affect the identities of each of the segments under the control of the BX-C. These mutations defined nine segment-specific functions. By genetic mapping, Ed Lewis discovered that these nine segments-specific functions are aligned along the chromosome in the same order as the segments they specify along the AP axis. This was the first identification of colinearity. Molecular analysis later revealed that the BX-C encoded only three, homeotic genes and that the genetically identified segment-specific functions were probably regulatory in nature. This was confirmed by antibody staining in mutant embryos. Antibody staining showed that *Ubx*, *abd-A*, and *Abd-B* are expressed in overlapping domains in the posterior half of the embryo (see also below). These expression patterns are intricate and finely tuned from one parasegment to the next (see for example **Figure 2**). By staining various mutant embryos it was shown that the segment-specific functions correspond to *cis*-regulatory regions that regulate the expression of *Ubx. abd-A,* or *Abd-B* in a parasegment-specific fashion. Thus the a*bx/bx* and *bxd/pbx cis-* regulatory regions direct *Ubx* expression in PS5 and PS6, respectively. Similarly the *iab-2* through *iab-4 cis*-regulatory regions direct the parasegment-specific expression patterns of *abd-A* in PS7, PS8, and PS9 (**Figures 1** and **2**; for review, see Maeda and Karch, 2006). And finally, the *iab-5* trough *iab-8 cis*-regulatory regions regulate *Abd-B* in PS10 to PS13, respectively. Thus, the collinearity that exists in flies extends beyond the genes themselves to the *cis*-regulatory elements that drive the Hox gene expression.

## *Antp* Ubx, abd-A, and *Abd*-B Hox GENES ARE EXPRESSED IN BROAD DOMAINS

Like in vertebrates, most *Drosophila* Hox genes are expressed in broad domains along the AP axis. This is the case for the *Antp* gene that specifies the identity of PS4. While its segmental specification role is restricted to this single parasegments, *Antp* remains expressed in all the more posterior parasegments, until PS12 (Hafen et al., 1984) Similarly, the *Ubx* gene that specifies PS5 and PS6 identities remains expressed up to PS12 (White and Wilcox, 1984; Akam and Martinez-Arias, 1985; Beachy et al., 1985). Finally, *abd-A* that specifies PS7 to PS9 remains expressed up to PS12 (Karch et al., 1990; Macias et al., 1990; see **Figure 2B**). Thus these three Hox genes remain expressed posterior to the parasegments they specify respectively (though, in each parasegment, expression is limited to a subset of cells, see below).

*Abd-B* organization is a bit more complex than its counterparts of the BX-C, *Ubx,* and *abd-*A. While *Abd-B* is also expressed in a broad domain (**Figure 2A**), it is expressed as a parasegmental step-wise gradient and plays a visible specification role in all the parasegments where it is expressed (from PS10/A5 to PS13/A8; Kuziora and McGinnis, 1988; Celniker et al., 1990; Delorenzi and Bienz, 1990). Also, there is an alternatively spliced, truncated form of *Abd-B* originating from upstream promoters (B, C, and γ). This alternatively spliced isoform produces a truncated protein called *Abd-B^r^*, which is expressed in PS14 (see **Figures 2D, 4E, 5B, 6A, 8A** or **9A**) and where it plays a role in specifying PS14 identity (see below for more details).

## TRANSCRIPTIONAL POSTERIOR DOMINANCE OF Hox GENES

Looking at the overall parasegment-specific expression pattern of *Ubx* and *abd-A*, or that of* abd-A* and *Abd-B* (**Figure 2**), their respective expression domains appear complementary to each other. These complementary appearances result from a general rule referred as to as “posterior dominance” in which a posterior Hox gene represses the expression of the immediately adjacent anterior Hox gene. For instances, *abd-A* represses *Ubx* in PS7 to PS12 (Struhl and White, 1985), and *Abd-B* represses *abd-A* in PS10 to PS13 (Karch et al., 1990). It should be noted that *abd-A* repression is not easily visible in PS10, PS11, and PS12, as *Abd-B* is expressed in only a few cells in these parasegments. A similar negative, *trans*-regulatory interaction exists between *Ubx* and *Antp*, the Hox gene responsible for PS4 specification. In this case, *Ubx* is known to repress *Antp* (Hafen et al., 1984; Carroll et al., 1986).

**FIGURE 3:**
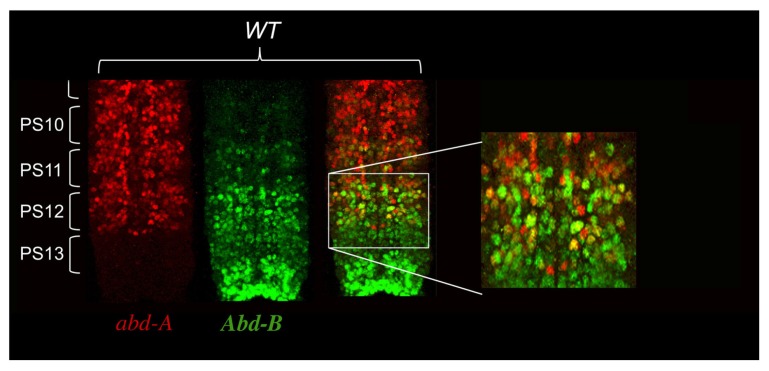
***abd-A* and *Abd-B* are both co-expressed in some cells of the central nervous system.** CNS of stage 15 embryos stained for *abd-A* (red) and *Abd-B* (green) were dissected and mounted on a slide with anterior on top. Parasegments boundaries are shown. Note the presence of neurons in PS10 to PS12 expressing both proteins as seen by the yellow color. Often the neurons expressing high level of *abd-A* also express *Abd-B*. (original observation published in Gummalla et al., 2012).

As a result of these negative cross-regulatory interactions, each parasegement is a mosaic of cells expressing different combinations of Hox genes. In Peifer et al. (1987) proposed that parasegmental identity was the readout of the unique mosaicism in each parasegments. This model predicts that each cell within a parasegment expresses a single Hox gene. In order to test his hypothesis, we carefully reexamined Hox gene expression in the *Drosophila* embryo using confocal microscopy analysis with antibodies directed against *Ubx*, *abd-A*, and *Abd-B*. The general rule that a given Hox gene represses expression of the immediately anterior expressed Hox gene appears mostly true. However, there is a notable exception with *abd-A* and *Abd-B* in the central nervous system (CNS), where both proteins are found co-expressed in many cells (**Figure 3**). Interestingly, we often found that cells with the highest levels of Abd-A protein also express high levels of Abd-B protein (**Figure 3**).

## *Abd*-B DOES NOT REPRESS *abd*-A IN THE EMBRYONIC CENTRAL NERVOUS SYSTEM

**FIGURE 4:**
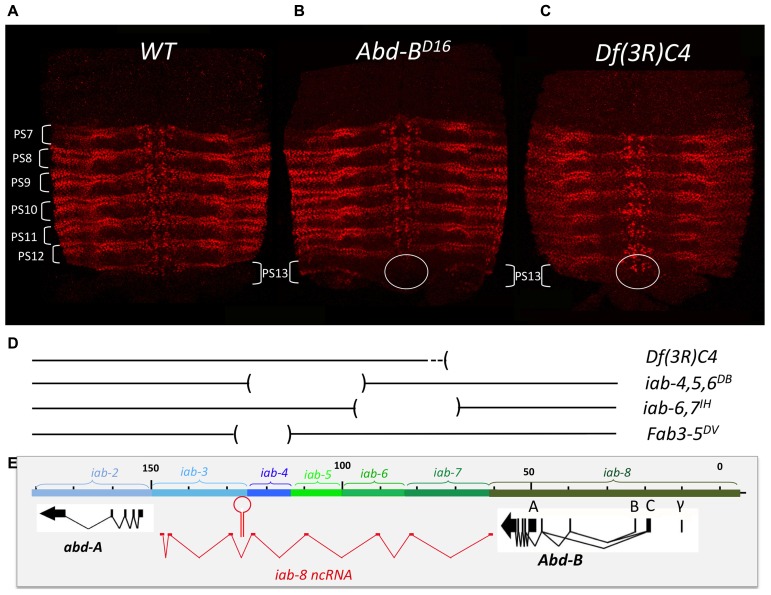
***abd-A* expansion in PS13 in *Abd-B* mutant context is restricted to the epidermis.** Pelts of stage 15 embryos stained for *abd-A* were prepared as in **Figure 1**. The WT expression pattern from PS7 to PS12 is shown in panel **A**. Panel **B** shows the pattern of *abd-A* expression in a homozygous *Abd-B^D16^* mutant embryo. Note the expansion of *abd-A* expression in PS13 in the epidermis. In the CNS, however, (circled) there is no expansion. Panel **C** shows a homozygous *Df(3R)C4* mutant embryo in which *abd-A* expansion in PS13 occurs in both epidermis and CNS (circled; original observation published in Gummalla et al., 2012). Panel **D** depicts the extend of the various deficiencies we used in our unsuccessful attempts to locate a second discrete repressive mechanism (see page 13). Panel **E**, same as panel **D** in **Figure 2**.

**FIGURE 5:**
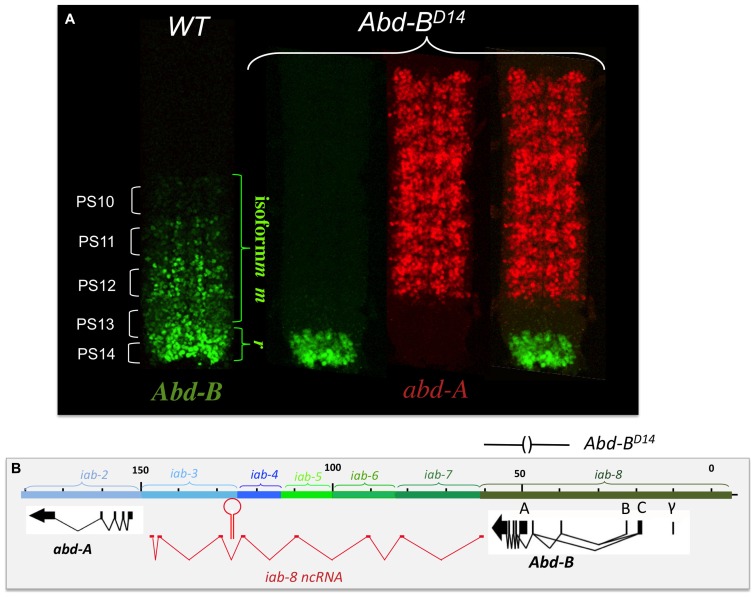
***abd-A*is still repressed in the CNS of *Abd-B*^D14^ mutant embryo.** *Abd-B^D14^* removes the promoter A of the *Abd-B* transcription unit (indicated above panel **B**). As the A promoter is regulated by the *iab-5*, *iab-6*, *iab-7*, and *iab-8* regulatory domains, there is no *Abd-B* expression in PS10 to PS13 (see panel **A**). In PS14, however, a truncated version of *Abd-B* (cross-reacting with the antibody) is expressed in PS14 from the B, C, and γ promoters (panel **A**). This result indicates the existence of alternate mechanism(s) (than *Abd-B* repression) to keep *abd-A* off in PS13 (original observation published in Gummalla et al., 2012).

**FIGURE 6:**
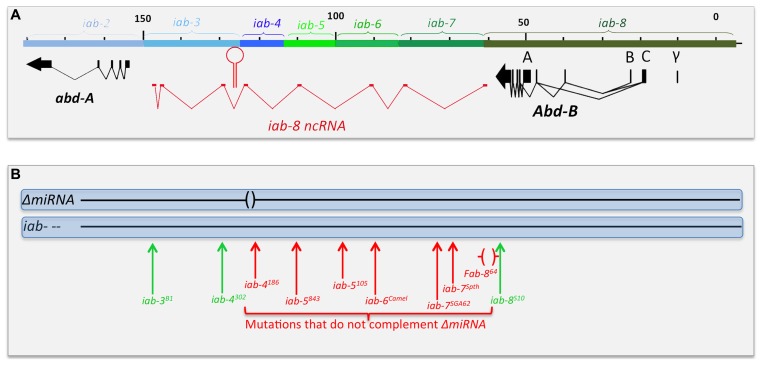
**Chromosomal breaks to the right of the miR-iab-8 fail to complement *ΔmiRNA*.** Panel **A** shows the genomic map of the abdominal region of the BX-C as described in **Figure 2**. Panel **B** symbolizes the two homologs chromosomes of heterozygotes between *ΔmiRNA* and various rearrangement breakpoints that disrupt the abdominal region of the BX-C. Breaks in red fail to complement the sterility phenotype of ΔmiRNA, while break in green are fully fertile over ΔmiRNA. The *Fab-8^64^* deletion removing the promoter of the iab-8 ncRNA is indicated by red brackets.

**FIGURE 7:**
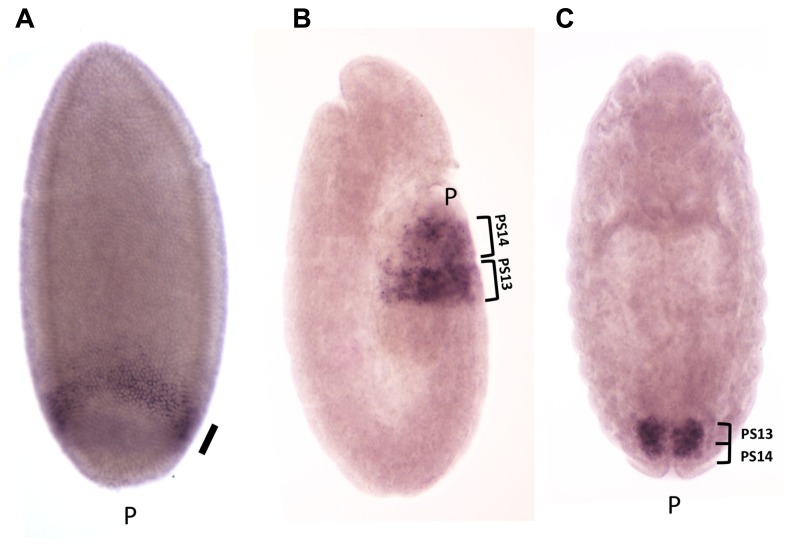
**Expression pattern of the *iab-8 ncRNA.*** Embryos were hybridized with a strand-specific probe derived from the *iab-6* region, to detect transcription in the same polarity than *abd-A* and *Abd-B*. Panel **A** shows an embryo 3 h after fertilization at the cellular blastoderm stage. **A** uniform band is visible at the posterior end of the embryo (shown by the thick oblique bar). At this stage, transient transcription from the *iab-6* regulatory regions is detectable in PS11 (oblique arrow). At the elongated germ band stage **(B)**, transcription is visible in the epidermis in PS13 and PS14. Panel **C** show a stage 15 embryo, after germ band contraction. Transcription is restricted to the CNS in PS13 and PS14 (original observation published in Bender, 2008).

**FIGURE 8:**
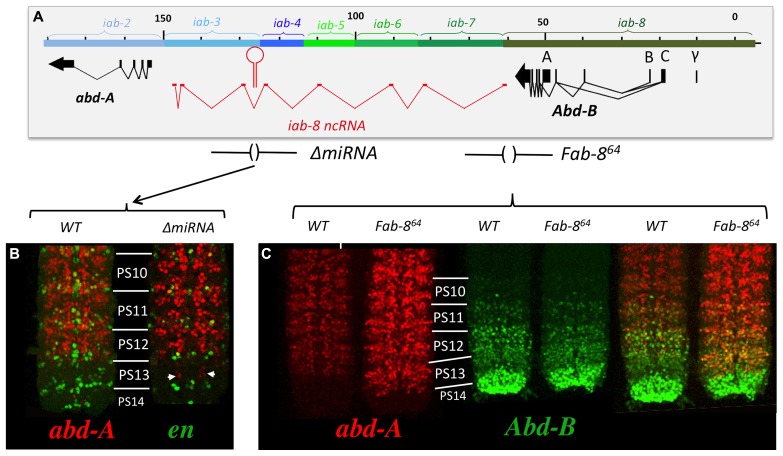
***abd-A* is only de-repressed in a few cells in PS13 in *ΔmiRNA.*** Panel **A** shows the genomic map of the abdominal region of the BX-C as described in **Figure 2** with the ΔmiRNA deletion drawn above. CNSs were dissected out from stage 15 embryos Note in panel **B** that *abd-A* is de-repressed in only few neurons in PS13. Panel **C** show the *abd-A*(red) and *Abd-B* (green) expression patterns in WT and *Fab-8^64 ^* homozygotes. Note the complete de-repression of *abd-A* in PS13 (original observation published in Gummalla et al., 2012).

The finding of cells expressing both *abd-A* and *Abd-B* contradicted the posterior transcriptional dominance rule of Hox genes as established by previous experiments. This prompted us to reexamine some of these experiments in more detail. Previously, it was shown that in the absence of *Abd-B* protein, *abd-A* protein becomes ectopically expressed in more posterior parasegments (Karch et al., 1990). This finding supported the idea that *Abd-B* and the posterior dominance rule restricted *abd-A* to more anterior abdominal parasegments. When we examined *Abd-B* null mutants in detail, we found that while we do indeed observe an extension of *abd-A* expression in PS13 in the epidermis, expression in the CNS remains unaffected (**Figure 4B**). This can, perhaps, be more easily seen in *Abd-B^D14^* mutants (*Abd-B^D14^*; **Figure 5**). As mentioned above, the *Abd-B* transcription unit displays some complexity, harboring multiple promoters (marked A, B, C, and γ; **Figures 1**–**8**; Zavortink and Sakonju, 1989; Boulet et al., 1991). Transcription initiating from the A promoter encodes the long isoform of the *Abd-B* protein referred as to the “m” isoform (for morphogenetic function; Casanova et al., 1986). The ABD-B m isoform is expressed from PS10/A5 to PS13/A8, thereby assigning identities to these parasegments/segments. Promoters B, C, and γ are only active in PS14. Splicing of these transcripts lead to the generation of a shorter isoform of *Abd-B* lacking the N terminal sequences of the m isoform. This shorter isoform is referred as to the “r” isoform (for regulatory function; Casanova et al., 1986; Kuziora and McGinnis, 1988; Boulet et al., 1991). In *Abd-B^D14^*, a deletion removes the “A” promoter along with the N-terminal coding sequences of the ABD-B m isoform (Karch et al., 1985; Zavortink and Sakonju, 1989). As a consequence, there is no detectable expression of *Abd-B* in PS10 through PS13 (**Figure 5**). In agreement with this observation, the few emerging escaper flies have their fifth through eighth abdominal segments transformed into the fourth abdominal segment (Karch et al., 1985). In PS14, however, note the presence of the truncated “r” ABD-B isoform that is encoded by transcripts initiating from the B, C, and γ promoters. While *Abd-B* is absent in PS10-13, there is no extension of *abd-A* expression in the CNS into PS13 in the context of the *Abd-B^D14^* mutant background (as illustrated by the gap between the red and green staining in **Figure 4**). This indicates that *Abd-B* is probably not responsible (or at least, not exclusively responsible) for *abd-A* repression in PS13.

We further confirmed this finding by asking if ectopic *Abd-B* could repress *abd-A* in the CNS. If PS13 like levels of *Abd-B* could repress *abd-A*, then ectopically activating *Abd-B* to PS13 levels in another PS, should repress *abd-A* expression. To do this, we used the *Fab-8^205^* mutation (Barges et al., 2000). *Fab-8^205^* is a mutation that removes a *cis*-regulatory domain boundary between *iab-7* and *iab-8*. Through a mechanism that is too complex to explain here, this deletion results in *iab-8*, normally driving PS13 levels of *Abd-B* expression, being activated in PS12. As expected of such a mutation, *Fab-8^205^* results in a homeotic transformation of PS12/A7 into PS13/A8. Staining of *Fab-8^205^* for *abd-A* showed normal levels of *abd-A* protein in PS12, indicating that PS13 levels of Abd-B cannot repress *abd-A* in the CNS(data not shown).

## *abd*-A DEREPRESSION IN MUTATIONS EFFECTING A LONG-NON-CODING RNA

Based on these results, two possibilities can be imagined to account for the lack of *abd-A* expression in PS13 of the CNS. The simplest possibility is that *abd-A* may not be expressed in PS13 simply because it is never turned on. This would imply that the *iab cis*-regulatory domains act differently on *abd-A* in the epidermis versus the CNS. Alternatively, the lack of *abd-A* in PS13 of the CNS could results from a different, not-yet-identified repressive mechanism.

**FIGURE 9:**
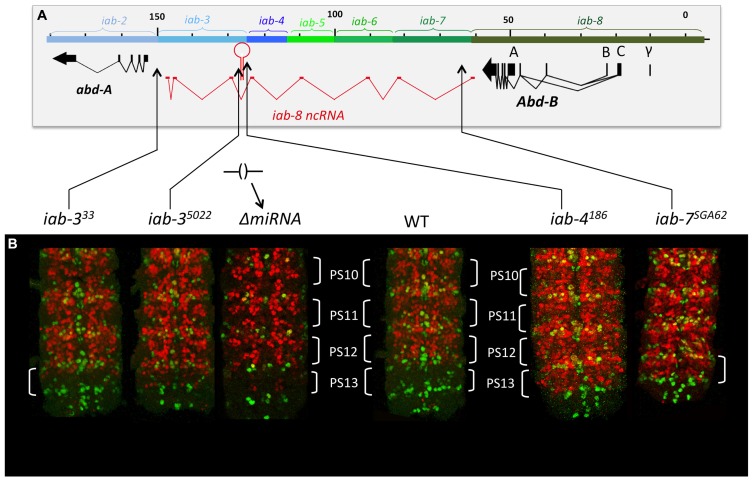
***abd-A* expression in the CNS in mutant that truncate the iab-8 ncRNA.** Panel **A**, show the molecular map of the abdominal region of the BX-C as in the figure above. The various rearrangement breaks truncating the iab-8ncRNA are shown below the map, along with the ΔmiRNA. Panel **B** show the posterior CNS of embryos that were stained for *abd-A* (red) and *engrailed* (*en*, green). The *engrailed* stripes mark each of the parasegments. Note that rearrangements disrupting the iab-8ncRNA upstream from miR-iab-8 lead to a complete de-repression of *abd-A* in the CNS in PS13 (*iab-6^186^, iab-7^SGA62^*). Rearrangements breaks disrupting the iab-8-ncRNA downstream from the site of miR-iab-8 result in only a partial de-repression of *abd-A* in PS13. A ΔmiRNA CNS is also shown for comparisons (original observation published in Gummalla et al., 2012).**

Mutation analysis points to the latter hypothesis as being correct. *Df(3R)C4* is a large deficiency that removes the entire *Abd-B* transcription unit as well as *iab-8* and about half of the of *iab-7* (**Figure 4D**). Staining for *abd-A* protein in *Df(3R)C4* embryos demonstrates that *abd-A* can be expressed in the CNS of PS13 (**Figure 4C**), suggesting that a repressive mechanism is involved in limiting *abd-A* expression. As we know *Abd-B* is not involved in this repression, we must assume that *Df(3R)C4* must delete additional sequences essential for the this second repressive mechanism. Previously, a large, 92 kb ncRNA spanning the intergenic region between *abd-A* and *Abd-B* was discovered emanating from a region in *iab-8* near the *Fab-8* boundary (see below and **Figures 2D, 4E, 5B, 6A, 8A**, and **9A**). We wondered if this long non-coding RNA (lncRNA), called the *iab-8*-ncRNA, could be involved in *abd-A* repression. As the promoter for the *iab-8*-ncRNA mapped to a region in *iab-8* just next to the *Fab-8* boundary, we examined *abd-A* expression in a larger *Fab-8* deletion* (Fab-8^64^*) that also removes the ncRNA promoter. Interestingly, we found that in *Fab-8^64^* mutants, we could see ectopic *abd-A* in PS13 even though *Abd-B* was expressed in both PS12 and PS13 at PS13 levels (**Figure 8C**). In fact, the levels of *abd-A* protein in PS13 resembled the levels of expression normally seen in PS12. Thus, these results pointed to the long *iab-8* ncRNA as the probable source of *abd-A* repression in PS13 of the CNS.

## THE iab-8 ncRNA TRANSCRIPTION UNIT AND THE miR-iab-8 GENE

The first evidence for the existence of a large transcription unit spanning the *abd-A/Abd-B* intergenic region arose with the emergence of *in situ* hybridization techniques. Already, Sanchez-Herrero and Akam (1989) noticed the presence of a signal at the posterior end of the embryos detected with many large genomic probes. Then, several studies reported similar embryonic expression patterns in the CNS and epidermis in PS13 and 14 with strand-specific probes detecting transcripts oriented from *Abd-B* toward* abd-A* (Bae et al., 2002; Drewell et al., 2002; Hogga and Karch, 2002; Rank et al., 2002; Schmitt et al., 2005). The similarity between the expression patterns reported in these various studies was evident, but it was only in 2008 that it became clear that they reflected the existence of a very large transcription unit active in PS13 and PS14 (Bender, 2008). In Bender (2008) used gene conversion to generate a surgical deletion of a miRNA located between *abd-A* and *Abd-B*. At the time, it was known that the miRNA was expressed from both DNA strands and were called miR iab3-4 and miR iab 4-3 respectively, based on the orientation of the transcription unit producing the miRNA. The deletion created was only 45 nucleotides long (henceforth called *ΔmiRNA*) to remove only the sequence encoding the two miRNAs. Although both miRNAs were predicted to target the *Ubx* and *abd-A* Hox genes, flies homozygous for the deletion did not harbor any segmental abnormalities, indicating that both miRNAs probably do not have a strict “homeotic function.” While the body structure and anatomy of these flies appeared completely normal, both females and males deleted for these miRNAs are sterile. However, this sterility does not seem to stem from a physical problem with their reproductive organs (gonads and/or the genitalia). Instead, the sterility phenotype present in *ΔmiRNA* flies seems to stem from a neuronal defect that makes them either unable to copulate (males) or unable to deposit eggs (females).

In as much as the miRNA gene is transcribed on both strands, Bender (2008) used a classical complementation test to determine if the sterility resulted from failure in the production of one or the other (or both) miRNA. Drawing from the vast collections of bithorax alleles that interrupt the chromosomal continuity of the abdominal region of the BX-C, he determined that a 65 kb-long region between the miRNAs and *Abd-B* was required for the production of the miRNA (**Figure 6B**), as any chromosomal break within this region (the red vertical arrows in **Figure 6B**) failed to complement *ΔmiRNA.* Breaks further to the left of the site of the miRNAs (**Figure 6B**) or to the right of *Fab-8^64^* (green vertical arrows) are fertile when in *trans* to *ΔmiRNA*. These observations indicate that the sterility phenotype is caused by loss of the miRNA produced from sense stand (relative to *abd-A* and *Abd-B* transcription) and define the region of DNA required for the production of this template RNA that spans from the region just downstream of the *Abd-B* transcription unit and extending to, at least, the site of the miRNA. As the position of the promoter lies within the *iab-8* regulatory domain, the transcript was named the *iab-8-*ncRNA and the miRNA was renamed miR-*iab-8* (Bender, 2008). RACE and RNAseq data later led to the precise definition of the *iab-8-*ncRNA as a 92 kb-long transcription unit spanning the entire *abd-A-Abd-B* intergenic region (Enderle et al., 2011; Graveley et al., 2011; Gummalla et al., 2012). Remarkably, the pri-miRNA transcript is spiced, with an exon derived from each of the *iab cis*-regulatory domains. A comparison with the genomic sequence data from 13 *Drosophila* species revealed that the transcript is conserved. Intriguingly, it is not the exonic sequences that are the most conserved, but the intron/exon junctions, as if it was the act of spicing that matters for the function of the *iab-8-*ncRNA. At present, there is no hint at the function of the spliced product or at the role of spicing in the function of the *iab-8-*ncRNA and/or miR-*iab-8*.

The expression pattern of the *iab-8-*ncRNA (and thus miR-*iab-8*) is consistent with the location of the promoter in *iab-8*, which controls the expression of *Abd-B* in PS13. The *iab-8-*ncRNA transcripts first appear at the posterior end of the embryo 3 h after fertilization, at the cellular blastoderm stage (**Figure 7A**). When the first signs of segmentation are visible (during germband elongation, **Figure 7B**), expression is restricted to PS13 and PS14 and mostly visible in the epidermis. After germband retraction, at the developmental stage where the nerve chord become visible, expression decays rapidly in the epidermis and become predominantly expressed in the CNS in PS13 and PS14, where it remains until for some time (**Figure 7C**). In fact, PS13/14 expression can even be seen in the CNS of third instar larvae (unpublished).

## *mi*R-iab-8 REPRESSES *abd*-A IN THE CNS IN PS13, BUT THIS IS NOT THE WHOLE STORY

Several features of miR-*iab-8* made it the prime candidate to be the repressor of *abd-A* expression in PS13 of the CNS. First, bioinformatics analysis predicted *abd-A* as a probable target of miR-*iab-8.* Second, it was strongly expressed in the cells where *abd-A* is repressed (PS13 of the CNS). Third, deletion of its promoter leads to a strong derepression of abd-A. And finally, reporter and ectopic expression studies showed that the *abd-A* 3′ UTR could in fact be targeted by the miRNA for translational repression (Stark et al., 2008; Tyler et al., 2008). Based on these findings, it seemed obvious that deletion of the miRNA would lead to *abd-A* derepression.

Examining *abd-A* expression in the CNS of *ΔmiRNA* mutant embryos showed that there is indeed a misexpression of *abd-A* in animals lacking miR-*iab8*. Surprisingly, however, this misexpression is limited to only a few neurons (**Figure 8B**). Furthermore, the misexpression appears stochastic as the pattern of derepression varies between different nerve chords. This observation was unexpected as the deletion of the promoter caused much more drastic derepression (**Figure 8C**). Based on this result, we hypothesized the existence of a second, partially redundant mechanism involving the *iab-8-*ncRNA to keep *abd-A* repressed.

## SEARCHING FOR A SECOND REPRESSION MECHANISM

As mentioned earlier, deletion of the *iab-8-*ncRNA promoter resulted in a complete derepression of *abd-A* in PS13. We used this phenotype to map additional elements in the *iab-8-ncRNA* that were important for *abd-A* repression. To do this, we first stained embryos, homozygous for various internal deficiencies in the *iab-8-*ncRNA sequence, thinking that if something like a second miRNA existed in the transcript, we might be able to identify it in this manner (Mihaly et al., 2006). Unfortunately, all deficiencies tested, with the exception of *Fab-3-5^DV^*, which removes the iab-8-miRNA, show no phenotype in this assay (see **Figure 4D** for the extend of the deficiencies). It must be noted, however, that while most of the ncRNA sequence has been tested by deletion analysis, we have no deficiencies spanning the 3′ end of the RNA (a region of about 15 kb) that do not also remove the *abd-A* promoter.

Therefore, to continue this analysis, we next decided to stain embryos from flies homozygous for chromosomal rearrangements that break the continuity of the *iab-8-*ncRNA. Using these lines, we found that all breaks lying in between the miRNA and its promoter showed complete derepression of *abd-A* in PS13 of the CNS (**Figure 9**). For example, break *iab-4^186^*, which breaks just upstream if the miRNA, shows a complete derepression of *abd-A* in PS13 of the CNS, much like an *iab-8-*ncRNA promoter deletion. Meanwhile, breaks lying between the miRNA and its 3′ end, which presumably still make the miRNA, showed a much milder, but visible derepression of *abd-A* in PS13 (**Figure 9**). This phenotype was reminiscent of *ΔmiRNA* embryos (see, for example *iab-3^5022^* in **Figure 9**). Based on the 3′-most rearrangement that causes a derepression of abd-A, we can limit the area where this second element must lie to a sequence of, at most, 5 kb (due to the resolution of the mutation mapping). This area contains two exons of the lncRNA and lies just 5′ to the *abd-A* transcriptional start site.

As stated above, we have no deficiencies covering most of this area that do not also remove the *abd-A* promoter. Therefore, we have had difficulty identifying the exact mechanism of this repression. However, a number of observations make us believe that the second mechanism does not involve a diffusible molecule, but simply depends on the transcription of the region around the *abd-A* promoter. First, no miRNAs have been predicted bioinformatically, or found from any miRNA screens, derived from the area in question. Second, although the transcript is spliced and polyadenylated, no known polypeptides are encoded by this transcript. Here, it must be noted that our colleague, Bender (2008) has studied the resulting cDNA from the spliced *iab-8-*ncRNA transcript in the fly. While he has found a conserved sequence in the eighth exon that could encode a micropeptide (Gummalla et al., 2012), overexpression of the *iab-8* cDNA has no affect on *abd-A* expression.

**FIGURE 10:**
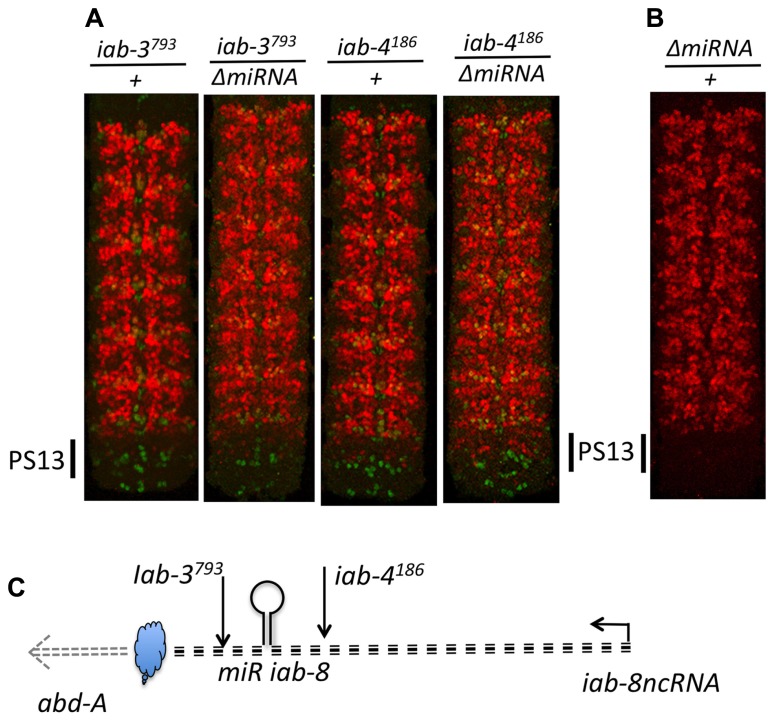
**Haplo-insufficiency of breaks disrupting the iab-8 ncRNA.** Panel **A** show CNSs stained for *abd-A* (red) and *engrailed* (green) from embryos heterozygous for mutations disrupting the iab-8ncRNA. Panel **B** dispalys a CNS from a heterozygous ΔmiRNA/+ embryo. Note that while one dose of miR-iab-8 is sufficient to keep *abd-A* repressed in PS13 **(B)**, de-repression of *abd-A* in PS13 is observed in each of the four genotypes displayed in panel **A**. Panel **C** summarizes the relative positions of the *trans*-acting repression mechanism (miRiab-8) and *cis*-acting repression mechanism symbolized as a cloud. The level of de-repressions depends on the position of the disrupting break (upstream or downstream of miRiab-8). De-repression increases when the disrupting break is over ΔmiRNA. In *iab-4^186^/ΔmiRNA* PS13 *abd-A* expression reaches a level as if only one of the two homologs produces *abd-A.*

Based on our mapping experiments, we know that the second repressive function must be located in the last ~5 kb of the *iab-8-*ncRNA. Much of this sequence makes up the final two exons of the *iab-8-*ncRNA, whose spliced product seems to play no role in *abd-A* regulation. As this region also includes the upstream promoter area of *abd-A*, we wondered if the act of transcribing this area could provide the repressive function. This was a difficult thing to test because of the lack of genetic tools in the area. Still, we thought about what such a mechanism would imply. We reasoned that diffusible molecules should work both in *cis* and in *trans*, meaning that if one copy of the element is mutated, the product of the other copy of the element should be able to compensate for its loss, since it is a diffusible molecule. Indeed, loss of one copy of the *iab-8-*miRNA shows no effect on *abd-A* expression (it is recessive). However, if the mechanism was transcription across the *abd-A* promoter, then this mode of repression should only worked in *cis*, as the wild-type copy of the element on one chromosome should not be able to compensate for its loss on the other. We tested this by staining heterozygous rearrangement break mutants whose breaks were downstream of the miRNA. In all of the lines previously shown to derepress *abd-A* as homozygotes, we observed weaker but still noticeable derepression of *abd-A* as heterozygotes (**Figure 10**). The fact that a deficiency that removes the entire BX-C (including the *abd-A* and the *iab-8-*ncRNA) does not show a similar phenotype (Gummalla et al., 2012) means that this derepression is not due to simple haploinsufficiency for this second element and points to a *cis*-dominant effect, consistent with our model that transcription across the *abd-A* promoter causes *abd-A* repression.

This type of repressive mechanism is generally called transcriptional interference. Although some instances of this phenomenon have been reported in metazoans, it has mostly been observed in yeast where one gene is inhibited by the transcription of its promoter region by a polymerase transcribing from an upstream gene (Greger and Proudfoot, 1998; Martens et al., 2005; Kim et al., 2012). This is very similar to the situation we observe at the *abd-A* locus, where the *iab-8-*ncRNA, though its promoter lies 93 kb away, is transcribed across the intervening sequence until within about 1 kb of the *abd-A* transcriptional start site. Though we have not proved this, we imagine that this transcription would then prevent promoter proximal enhancer elements from initiating transcription at the *abd-A* promoter.

## A RETURN TO POSTERIOR DOMINANCE AND EVOLUTIONARY CONSIDERATIONS

We started this review by explaining how *abd-A* regulation in the CNS seems to break the posterior dominance rule of the *Drosophila* Hox genes. Now, with this new data, we realize that this may not be the case. The transcriptional control of *abd-A* by the *iab-8-*ncRNA can simply be viewed as a modified example of posterior dominance. In this case, the repression occurs not through a transcription factor, but through two, completely different mechanisms: a miRNA-based repression mechanism and what is most likely a transcriptional interference-based repression mechanism. If we think of the *iab-8-*ncRNA as a Hox complex “gene,” then a more-posterior “gene” is still inhibiting a more anterior Hox gene, which fits with the posterior dominance rule.

The transcriptional interference model also provides another reason to explain the clustering of Hox genes in the fly. Transcriptional interference relies on having two genes in close proximity, so that the transcription of one interferes with the promoter of the other. Here, this seems to have been accomplished by the transcription of a lncRNA interfering with the promoter of the *abd-A* gene. The fact that loss of the ncRNA causes sterility and that it is initiated from a promoter in the *iab-8cis*-regulatory domain (which controls *Abd-B* expression) means that there will be selective pressure to keep the *abd-A* and *Abd-B* genes clustered.

But why create such a complex mechanism to control *abd-A* expression in PS13 and 14 of the CNS? Although we cannot truly answer this question, we can provide some thoughts on the issue. First, we must assume that, in the CNS, there is a reason to eliminate the standard cross-regulatory interactions between *Abd-B* and *abd-*A to allow co-expression of the two Hox genes in the same cell. While fate mapping work has been extensively done in the CNS, we have not identified all of the neurons that express *abd-A*, or *Abd-B* or both, to know if the combinatorial expression of Hox genes leads to modification of cell fate. However, having said that, we do have some indication that co-expressing at least some Hox genes might affect cell viability. Work from the lab of Alex Gould showed that expression of *abd-A* in larval abdominal neuroblasts was required for the cessation cell division and eventual apoptosis of these cells (Bello et al., 2003). This work stemmed from the idea that there must be something to control neuroblast division in the brain, and from the initial observation that the there were ~10× fewer neuroblasts in the abdominal segments than in the thoracic segments. Based on the abdominal localization of this phenomenon, Bello et al. (2003) asked if *abd-A* could mediate this loss of neuroblasts. Their experiments showed both that the loss of *abd-A* in abdominal neuroblast led to an increase in the pool of neuroblasts and that the ectopic expression of *abd-A* in thoracic neuroblasts led to a decrease in the pool of neuroblasts. These phenotypes eventually could all be attributed to a pulse of *abd-A* expression during the third instar stage that caused the neuroblasts to undergo apoptosis. As the loss of neuroblasts affects A1–A7, it seems likely that *abd-A* and *Abd-B* might have to be expressed in the same cell to have this phenomenon occur in A5–A7.

Next, we must ask why this new type of regulation happens in the CNS. As it turns out, regulation by miRNAs may be a common feature for neuronal genes in *Drosophila*. Work by the Levine lab has shown that the function of the common pan-neuronal gene ELAV is to bind to the 3′ UTR sequences of certain transcripts and to prevent normal polyadenylation. The result of this activity is the extension of 3′ UTR sequences for many neuronal genes (Hilgers et al., 2011, 2012). In agreement with this finding it has been long known that many Hox genes with extended 3′ UTR (*Antp, Ubx, abd-A*, and *Abd-B*) are specifically expressed in the nervous system (Garber et al., 1983; Scott et al., 1983; Akam and Martinez-Arias, 1985; Kuziora and McGinnis, 1988; O’Connor et al., 1988). Supporting this work, the lab of Claudio Alonso has recently shown that many Hox genes, including *abd-A*, possess CNS-specific 3′ UTR extensions. The result of these extensions is often an increase in the number of miRNA target sites. In the case of *abd-A*, the extension adds two additional targets for the *iab-8*-miRNA. Alonso and colleagues propose that these 3′ extensions could indicate a need to lose miRNA regulation in the epidermis or a need to augment miRNA regulation in the CNS (Thomsen et al., 2010). The fact that the *iab-8*-ncRNA is expressed primarily in the CNS would definitely support the latter hypothesis with regards to *abd-A* regulation.

Lastly, we must discuss why such a long transcript has been conserved to perform these functions when a much smaller transcript might be able to do the same. Indeed a transcript starting just upstream of the miRNA could, if expressed in the right place, perform the same function. We know, for example, that artificially starting a transcript downstream of the actual *iab-8*-ncRNA promoter can inhibit *abd-A* expression in anterior segments (Gummalla, 2011). However, if the *iab-8*-ncRNA is required only in the posterior parasegments, then how could such a smaller RNA be expressed only in PS13 and 14 within the context of a more-anterior *cis*-regulatory domain. Although gene regulation in the BX-C is a little too complex to explain in this review, we can say that, in general, promoters located in a specific *cis*-regulatory domain, gain regulation by that *cis*-regulatory domain. Thus, a promoter located in *iab-4* would probably be expressed in a pattern driven by the *iab-4cis*-regulatory domain (meaning that expression would start in PS9/A4) and would be expressed too anterior to be viable. Of course, one could simply imagine the cells of the CNS making a specific transcription factor or miRNA from another locus to inhibit *abd-A* expression in certain places, but then the issue becomes a matter of cost from where the system originated. Given that the fly has a system to elongate neuronal transcripts to provide more miRNA targets, and has a perfect place to obtain PS13 and 14 expression, we imagine that it was simpler to evolve the current ncRNA system than a secondary repressor. Given the large amount of ncRNAs currently being found in the cells of most organisms, it now seems clear that the energetic cost of transcription is probably not prohibitively high.

But this all assumes that transcriptional interference was added after the other mechanisms of Hox gene repression. This is still far from clear. It is possible that the first Hox genes were regulated by transcriptional interference. This is not an absurd notion to entertain. We know that the Hox genes were probably derived from tandem duplication events. Based on the similarity in construction of different *cis*-regulatory domains it seems likely that they too were made by duplication events happening later in evolution of an ancestral *cis*-regulatory region. Thus, the ancestral Hox complex contained just two very similar Hox genes, each probably controlled by small *cis*-regulatory domains. Each gene would probably express in a very similar pattern, having been duplicated from the same gene. Assuming a perfect duplication event, then the only differing feature with regards to these genes would be a slight difference in location on the chromosome and their neighboring genes. One can therefore imagine that if the 5′ gene could interfere with the transcription of its downstream brother, then this could have been one of the first events differentiating the two genes and allowing divergent functions to evolve.

## Conflict of Interest Statement

The authors declare that the research was conducted in the absence of any commercial or financial relationships that could be construed as a potential conflict of interest.

## Footnotes

1 An explanation of some *Drosophila* nomenclature. The segmental boundaries visible in the adult fly do not correspond to the reiterated units that form during the early stages of embryogenesis. In embryogenesis, the embryos is subdivided into units that are slightly shifted relative to the adult segments. These units are called parasegments, with one parasegement being composed of cells giving rise to the posterior part of one adult segment and the anterior part of the next segments (Martinez-Arias and Lawrence, 1985). For example, parasegment 5 (PS5) is makes up of the posterior part of the second thoracic segment (T2) and the anterior part of the third thoracic segment (T3). It is for this reason that we will generally refer to embryonic patterns in parasegmental nomenclature and adult patterns in segmental nomenclature. For the abdominal segments however, as their posterior compartment is not visible in adult fly (being folded under the anterior half) there is a fairly direct correlation between parasegmental and visible segmental borders. Thus, for the sake of simplicity, we will often simply refer both parasegments and segments simultaneously (i.e., the second abdominal segment will be referred to (A2/PS7).
